# Plasma BDNF Levels Following Transcranial Direct Current Stimulation Allow Prediction of Synaptic Plasticity and Memory Deficits in 3×Tg-AD Mice

**DOI:** 10.3389/fcell.2020.00541

**Published:** 2020-07-03

**Authors:** Sara Cocco, Marco Rinaudo, Salvatore Fusco, Valentina Longo, Katia Gironi, Pietro Renna, Giuseppe Aceto, Alessia Mastrodonato, Domenica Donatella Li Puma, Maria Vittoria Podda, Claudio Grassi

**Affiliations:** ^1^Department of Neuroscience, Università Cattolica del Sacro Cuore, Rome, Italy; ^2^Fondazione Policlinico Universitario A. Gemelli IRCCS, Rome, Italy

**Keywords:** Alzheimer’s disease, blood biomarkers, BDNF, neuroplasticity, personalized medicine, tDCS

## Abstract

Early diagnosis of Alzheimer’s disease (AD) supposedly increases the effectiveness of therapeutic interventions. However, presently available diagnostic procedures are either invasive or require complex and expensive technologies, which cannot be applied at a larger scale to screen populations at risk of AD. We were looking for a biomarker allowing to unveil a dysfunction of molecular mechanisms, which underly synaptic plasticity and memory, before the AD phenotype is manifested and investigated the effects of transcranial direct current stimulation (tDCS) in 3×Tg-AD mice, an experimental model of AD which does not exhibit any long-term potentiation (LTP) and memory deficits at the age of 3 months (3×Tg-AD-3M). Our results demonstrated that tDCS differentially affected 3×Tg-AD-3M and age-matched wild-type (WT) mice. While tDCS increased LTP at CA3-CA1 synapses and memory in WT mice, it failed to elicit these effects in 3×Tg-AD-3M mice. Remarkably, 3×Tg-AD-3M mice did not show the tDCS-dependent increases in pCREB^*Ser133*^ and pCaMKII^*Thr286*^, which were found in WT mice. Of relevance, tDCS induced a significant increase of plasma BDNF levels in WT mice, which was not found in 3×Tg-AD-3M mice. Collectively, our results showed that plasticity mechanisms are resistant to tDCS effects in the pre-AD stage. In particular, the lack of BDNF responsiveness to tDCS in 3×Tg-AD-3M mice suggests that combining tDCS with dosages of plasma BDNF levels may provide an easy-to-detect and low-cost biomarker of covert impairment of synaptic plasticity mechanisms underlying memory, which could be clinically applicable. Testing proposed here might be useful to identify AD in its preclinical stage, allowing timely and, hopefully, more effective disease-modifying interventions.

## Introduction

Alzheimer’s disease (AD) is a progressive neurodegenerative disorder responsible for the most common form of dementia. To date, therapeutic interventions against AD failed most likely because of late treatment initiation, i.e., when brain function and structure are already irreversibly damaged. Several lines of evidence suggest that pathogenic mechanisms of AD may affect the brain in the dark for many years owing to the brain’s ability to cope with failures exploiting the so-called “cognitive reserve.” Compensatory mechanisms can stave off neurodegeneration symptoms maintaining memory encoding for long time, and exhaustion of such brain ability may mark AD onset (Merlo et al., 2019). Thus, one primary goal is to detect preclinical AD, inasmuch as therapeutic interventions may have a higher success probability. Furthermore, some signs and symptoms, which manifested at early AD stages (e.g., depressive and cognitive symptoms in the measure of semantic memory and conceptual formation), are sometimes not recognized and/or mistaken for symptoms of other pathologies (Bature et al., 2017). This further stresses the need of reliable disease biomarkers, which may help early AD diagnosis.

Cognitive decline in AD is linked to pathological accumulation of amyloid-beta (Aβ) and Tau proteins and their aggregation in brain regions which are essential for memory encoding and storage, such as the medial temporal lobe and related cortical areas (Serrano-Pozo et al., 2011; Bloom, 2014). Striking evidence from preclinical studies indicates that both Aβ and Tau have detrimental effects on molecular machinery of synapses, ultimately leading to decreased hippocampal long-term potentiation (LTP), a cellular correlate of memory (Irvine et al., 2008; Kopeikina et al., 2012; Ripoli et al., 2014; Fá et al., 2016; Puzzo et al., 2017; Gulisano et al., 2018a, b). However, decreased synaptic plasticity, similarly, to memory impairment, is manifested when the pathology has already developed. Molecular pathways, underlying synaptic plasticity, potentially deregulated or vulnerable in the pre-symptomatic stage, might provide early biomarkers to predict the onset and/or progression of the disease.

Recent studies, including ours, have shown that molecular determinants of synaptic plasticity, including brain-derived neurotrophic factor (BDNF), phosphorylation of CREB at Ser133 (pCREB^*Ser133*^), calcium-calmodulin kinase II (CaMKII) at Thr286 (pCaMKII^*Thr286*^) and AMPA receptor GluA1 subunit at Ser831 (pGluA1^*Ser831*^), are engaged and boosted by transcranial direct current stimulation (tDCS) – a non-invasive neuromodulatory technique – resulting in increased LTP and enhanced cognitive or motor functions, depending on the stimulated brain area (Ranieri et al., 2012; Rohan et al., 2015; Podda et al., 2016; Kim et al., 2017; Paciello et al., 2018; Stafford et al., 2018; Barbati et al., 2019; Yu et al., 2019; Kronberg et al., 2020).

We hypothesized that tDCS might differentially impact LTP and memory in 3×Tg-AD mice, a common model of AD, at a stage when the AD phenotype is not manifested yet (i.e., at 3 months of age, hereinafter referred to as 3×Tg-AD-3M mice) (Oddo et al., 2003; Stover et al., 2015; Belfiore et al., 2019), thus unveiling early dysfunction of synaptic plasticity mechanisms.

We found that tDCS failed to enhance LTP at CA3-CA1 synapses and memory in 3×Tg-AD-3M mice whereas it increased these parameters in age-matched wild-type (WT) mice. Of note, 3×Tg-AD-3M mice did not show increased pCREB^*Ser133*^, pCaMKII^*Thr286*^, and BDNF following tDCS, suggesting that these molecular changes could serve as novel early biomarkers for AD. Remarkably, BDNF responsiveness to tDCS was assessed in blood samples, providing an easy-to-detect and low-cost biomarker.

## Materials and Methods

### Animals

Data of male triple transgenic AD (3×Tg-AD) mice, harboring the Swedish human APP, presenilin M146V and tauP301L mutations (Oddo et al., 2003) were compared to C57BL/6 wild-type (WT) mice (Li et al., 2018; Chakroborty et al., 2019; Joseph et al., 2019). The colonies were established in-house at the Animal Facility of the Università Cattolica from breeding pairs purchased from the Jackson Laboratory. The study was performed on 3-month-old (3M) 3×Tg-AD and WT mice (*n* = 78 and *n* = 88, respectively). Seven-month-old (7M) 3×Tg-AD mice and aged-matched WT mice (*n* = 21 each group) were also tested to validate the time course of AD phenotype in terms of synaptic plasticity and memory impairment in our experimental conditions. The animals were housed under a 12 h light-dark cycle at a controlled temperature (22–23°C) and constant humidity (60–75%).

### Ethics Statement

All animal procedures were approved by the Ethics Committee of the Catholic University and were fully compliant with guidelines of the Italian Ministry of Health (Legislative Decree No. 26/2014) and European Union (Directive No. 2010/63/UE) legislations on animal research. All efforts were made to minimize the number of animals used and their suffering.

### Electrode Implantation and tDCS Protocol

TDCS over the hippocampus was delivered using a unilateral epicranial electrode arrangement as previously described (Podda et al., 2016; Barbati et al., 2019). The active electrode consisted of a tubular plastic cannula (internal diameter 3.0 mm) filled with saline solution (0.9% NaCl) just prior to stimulation; the counter electrode was a conventional rubber-plate electrode surrounded by a wet sponge (5.2 cm^2^) positioned over the ventral thorax. The center of the active electrode was positioned on the skull over the left hippocampal formation 1 mm posterior and 1 mm lateral to the bregma (Franklin and Paxinos, 1997). A unilateral arrangement was chosen, as in our previous study, to reduce the electrode contact area and to prevent currents bypassing the two juxtaposed epicranial electrodes, which might occur using a bipolar configuration. Stimulation of the left side was preferred since experimental evidence suggests that long-term memory processing are strictly dependent on this hemisphere (Shipton et al., 2014). This electrode montage was previously shown to target the hippocampus causing neurophysiological, behavioral and molecular changes all related to this brain structure. Furthermore, no changes in BDNF levels were detected in non-stimulated areas such as the cerebellum, and tDCS of the motor cortex caused no changes in the hippocampus (see details in Podda et al., 2016). For electrode implant, animals were anesthetized by an intraperitoneal injection of a cocktail with ketamine (87.5 mg/Kg) and xylazine (12.5 mg/Kg) and temperature during surgery was maintained at 37°C. The scalp and underlying tissues were removed and the electrode was implanted using a carboxylate cement (3M ESPE, Durelon, 3M Deutschland GmbH, Germany). All animals were allowed to recover for 3–5 days before tDCS. During this period, as well as during the electrical stimulations, mice were placed in individual cages.

TDCS was applied to awake mice using a battery-driven, constant current stimulator (BrainSTIM, EMS, Italy). The current intensity was ramped for 10 s instead of switching it on and off to avoid a stimulation break effect.

A repeated tDCS protocol was used consisting in 3 single stimulation sessions (at a current intensity of 250 μA for 20 min, current density of 35.4 A/m^2^) once per day, on 3 consecutive days. According to clinical and brain slice conventions (Jackson et al., 2016; Rahman et al., 2017), we applied “anodal” tDCS corresponding to a positive electric field (positive electrode over the hippocampus). Electrode montage and current density were similar to those recently adopted for rodent models and close to the recommended safety limits in rodents (Rohan et al., 2015; Podda et al., 2016; Jackson et al., 2017; Paciello et al., 2018).

On the 3 consecutive days, tDCS was performed approximately at the same time (around 10 a.m.). No abnormal behaviors were observed related to the stimulation and no morphological alterations were found in brain tissues of mice subjected to tDCS.

Three-month-old WT and 3×Tg-AD mice were randomly assigned to the following experimental groups: (i) sham mice (sham-WT-3M, sham-3×Tg-AD-3M), which underwent the same manipulations as in the “real” stimulation condition, but no current was delivered; (ii) tDCS mice (tDCS-WT-3M, tDCS-3×Tg-AD-3M), which were subjected to repeated anodal tDCS. Different groups of mice were used for each experimental test.

### Electrophysiology

Field recordings were performed on hippocampal coronal slices (400 μm-thick) as previously described (Podda et al., 2008, 2016). Briefly mice were anesthetized by isoflurane inhalation (Esteve) and decapitated. The brain was rapidly removed and placed in ice-cold cutting solution (in mM: 124 NaCl, 3.2 KCl, 1 NaH_2_PO_4_, 26 NaHCO_3_, 2 MgCl_2_, 1 CaCl_2_, 10 glucose, 2 sodium pyruvate, and 0.6 ascorbic acid, bubbled with 95% O_2_-5% CO_2_; pH 7.4). Slices were cut with a vibratome (VT1200S) and incubated in artificial cerebrospinal fluid (aCSF; in mM: 124 NaCl; 3.2 KCl; 1 NaH_2_PO_4_, 26 NaHCO_3_, 1 MgCl_2_, 2 CaCl_2_, 10 glucose; 95% O_2_-5% CO_2_; pH 7.4) at 32°C for 60 min and then at RT until use. Slices were prepared ∼30 min after tDCS or sham stimulation protocol. Slices containing the stimulated hippocampus were used for subsequent analyses.

Slices were transferred to a submerged recording chamber and continuously perfused with aCSF (flow rate: 1.5 ml/min). The bath temperature was maintained at 30–32°C with an in-line solution heater and temperature controller (TC-344B, Warner Instruments). Identification of slice subfields and electrode positioning were performed with 4× and 40× water immersion objectives on an upright microscope (BX5IWI, Olympus) and video observation (C3077-71 CCD camera, Hamamatsu Photonics).

All recordings were made using MultiClamp 700B amplifier (Molecular Devices). Data acquisition and stimulation protocols were performed with the Digidata 1440A Series interface and pClamp 10 software (Molecular Devices). Data were filtered at 1 kHz, digitized at 10 kHz, and analyzed both online and offline.

Field recordings were made using glass pipettes filled with aCSF (tip resistance 2–5 MΩ) and placed in the stratum radiatum of the CA1 region. Field excitatory post-synaptic potentials (fEPSPs) were evoked by stimulation of the Schaffer collateral using a concentric bipolar tungsten electrode (FHC) connected to a constant current isolated stimulator (Digitimer Ltd.). The stimulation intensity that produced one-third of the maximal response was used for the test pulses and LTP induction. The fEPSP amplitude was measured from baseline to peak. The slope of the rising phase of the fEPSP was also calculated.

For LTP recordings, stable baseline responses were recorded to test stimulations (0.05 Hz for 10 min) and then a high-frequency stimulation (HFS) protocol was delivered (4 trains of 50 stimuli at 100 Hz, 500 ms each, repeated every 20 s). Responses to test pulses were recorded every 20 s for 60 min to assess LTP. LTP was expressed as the percentage of change in the mean fEPSP slope or peak amplitude normalized to baseline values (i.e., mean values for the last 5 min of recording before HFS, taken as 100%). HFS-elicited fEPSP changes in both amplitude and slope higher than 15% of baseline values were subjected to data analysis.

### Memory Test

Object recognition test, also known as novel object recognition (NOR) test and Morris water maze (MWM) test were used to assess non-spatial (i.e., recognition) and spatial memory, respectively. These tests were chosen since they are the most widely used and standardized tests of hippocampal-dependent forms of learning and memory (Vorhees and Williams, 2014; Cohen and Stackman, 2015).

Behavioral tests were carried out from 9 a.m. to 4 p.m. and data were blindly analyzed using an automated video tracking system (Any-Maze).

The NOR protocol lasted 3 consecutive days including a familiarization session, a training session and a test session. On the first day, animals were familiarized for 10 min to the test arena (45 cm×45 cm). On the second day (training session), they were allowed to explore two identical objects placed symmetrically in the arena for 10 min. On the third day (test session), a new object replaced one of the old objects. Animals were allowed to explore for 10 min and a preference index, calculated as the ratio between time spent exploring the novel object and time spent exploring both objects, was used to measure recognition memory (Fusco et al., 2019).

MWM was performed as previously described (Podda et al., 2014, 2016). A circular plastic pool (127 cm in diameter) filled with water colored with nontoxic white paint, to obscure the location of an hidden platform, was used as experimental apparatus. The pool was ideally separated into four equal quadrants (NE, corresponding to the target quadrant, SE, NW, and SW) and the platform (10 cm×10 cm) was placed at the center of the target quadrant. Visual cues were placed on the walls around the pool to orient the mice. Animals were trained for 4 days, six times a day and the probe test was administered 24 h after the last training day. Starting positions were varied daily and latencies to reach the platform were recorded. In the probe test, the platform was removed and time spent in the target quadrant was measured (60 s of test duration).

According to published protocols, the following exclusion criteria were applied: total exploration time < 5 s in the NOR test and floating behavior during training (i.e., not actively searching for the platform) in the MWM test. No animal met exclusion criteria and all results of behavioral studies were included in data analysis.

### Western Immunoblot

Total proteins were extracted from the stimulated hippocampus of control and tDCS-mice sacrificed 2 h after stimulation, using ice cold RIPA buffer [Pierce; 50 mM Tris, 150 mM NaCl, 1 mM EDTA, 1% DOC, 1% Triton X-100, 1% SDS, and 1× protease, phosphatase-1, and phosphatase-2 inhibitor cocktails (Sigma)]. Tissues were incubated for 15 min on ice with occasional vortexing and the lysate was spun down at 22,000×g for 15 min, 4°C, and 2 μl aliquot of the supernatant was assayed to determine the protein concentration (microBCA kit, Pierce). SDS-PAGE reducing sample buffer was added to the supernatant, and samples were heated to 95°C for 5 min. Protein lysates (40 μg) were loaded onto 10% or 8% Tris-glycine polyacrylamide gels for electrophoretic separation. Precision Plus Protein Dual Color Standards (Bio-Rad) were used as molecular mass standards. Proteins were then transferred onto nitrocellulose membranes at 330 mA for 2 h at 4°C in transfer buffer containing 25 mM Tris, 192 mM glycine and 20% methanol. Membranes were incubated for 1 h with blocking buffer (5% skim milk in TBST), and then incubated overnight at 4°C with primary antibodies directed against one of the following proteins: pCREB^*Ser133*^, CREB, pCaMKII^*Thr286*^, CaMKII, and GAPDH (Supplementary Table 1). After three 10 min rinses in TBST, membranes were incubated for 2 h at RT with HRP-conjugated secondary antibodies (Supplementary Table 1). The membranes were then washed, and the bands were visualized with an enhanced chemiluminescence detection kit (GE Healthcare, United Kingdom). Protein expression was evaluated and documented using UVItec Cambridge Alliance. Experiments were performed in triplicate.

### ELISA Measurements

Blood samples were collected from the retro-orbital plexus with sterile glass Pasteur pipettes. Samples were taken before and 1 week after tDCS. After centrifugation, plasma was separated and stored at −80°C until further use. Plasma levels of BDNF were determined using commercially available ELISA kits (Immunological Sciences). The assay was performed according to the manufacturer’s instructions on samples collected from 4 animals per group, and each sample was analyzed in duplicate.

### Statistical Analysis

Sample sizes were chosen with adequate statistical power (0.8) according to results of prior pilot data sets or studies, including our own using similar methods or paradigms. Sample estimation and statistical analysis were performed using the SigmaPlot 14.0 software. Data were first tested for equal variance and normality (Shapiro-Wilk test) and then the appropriate statistical tests were chosen. The statistical tests used [i.e., one-way ANOVA, one-way ANOVA for repeated measures (RM), Friedman RM ANOVA on Ranks, two-way ANOVA, two-way RM ANOVA] are indicated in the main text and in the corresponding figure legends for each experiment. *Post hoc* multiple comparisons were performed with Bonferroni correction. The level of significance was set at 0.05. Results are presented as mean ± SEM. Analyses were performed blinded.

## Results

### Characterization of Memory and Synaptic Plasticity Impairments in 3×Tg-AD Mice

The objective of the study was to test whether anodal tDCS can be exploited to unmask covert impairment of brain plasticity mechanisms in 3×Tg-AD mice before synaptic plasticity and memory deficits are clearly manifested in this AD mouse model, with the ultimate goal to identify early neurophysiological and molecular biomarkers allowing to predict disease onset.

Our first step was to characterize the time course of the 3×Tg-AD mouse phenotype in our experimental conditions, given that some variability has been reported in literature (Belfiore et al., 2019). Specifically, memory and LTP were assessed in 3 and 7 months old AD mice, chosen as putative pre-symptomatic and AD models, respectively. Different cohorts of mice were used for 3 and 7 months.

**FIGURE 1:**
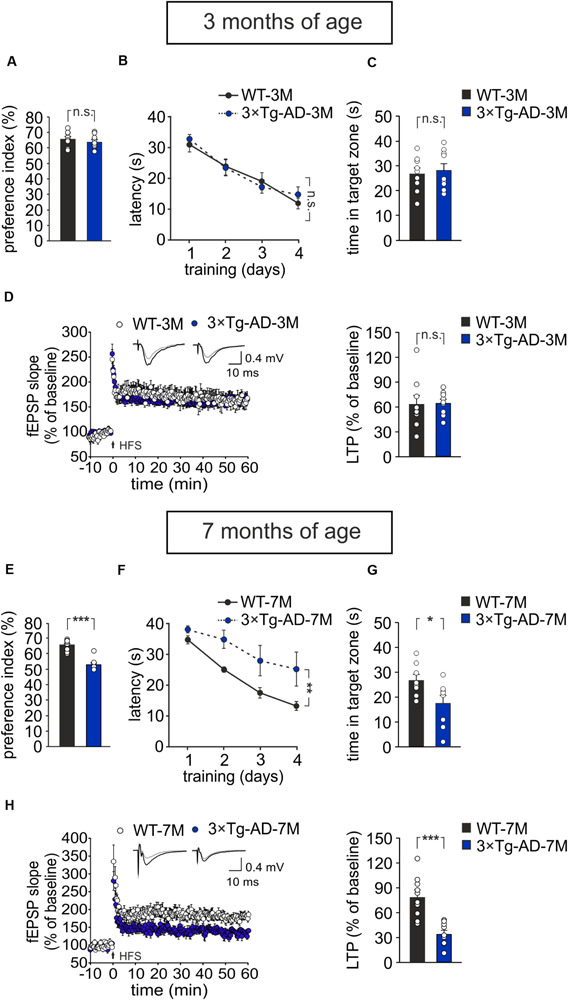
Age-dependent pathological memory and synaptic plasticity changes in 3×Tg-AD mice. **(A–D)** 3-month-old 3×Tg-AD mice did not differ from age-matched WT mice in: **(A)** the preference toward the novel object in the NOR test (*n* = 9 mice for each group; *P* = 0.40, one-way ANOVA); **(B)** the latency to platform in the training phase of the MWM test (*n* = 9 mice for each group; *P* = 0.73, two-way RM ANOVA) and **(C)** the time spent in the target quadrant during the probe test performed on day 5 of MWM (*P* = 0.66, one-way ANOVA); **(D)** the magnitude of LTP at hippocampal CA3-CA1 synapses (*n* = 9 slices from 5 3×Tg-AD-3M mice; *n* = 9 slices from 6 WT-3M mice; *P* = 0.89, one-way ANOVA). Time course shows LTP at CA3-CA1 synapses induced by HFS (4 trains of 50 stimuli at 100 Hz for 500 ms repeated every 20 s) delivered at time 0 (arrow). Results are expressed as percentages of baseline fEPSP slope (= 100%). Insets show representative fEPSPs at baseline (gray line) and during the last 5 min of LTP recording (black line). Bar graphs compare LTP observed during the last 5 min of recording. **(E–H)** Compared to aged-matched WT mice, 7-month-old 3×Tg-AD mice showed significant decreases in: **(E)** preference index in the NOR test (*P* < 0.001); **(F)** latency to platform in the training phase of the MWM test (*n* = 8 mice for each group; *P* = 0.009, two-way RM ANOVA) and **(G)** time spent in the target quadrant during the probe test of MWM (*P* = 0.032, one-way ANOVA); **(H)** LTP (*n* = 10 slices from 5 3×Tg-AD-7M mice; *n* = 10 slices from 5 WT-7M mice, *P* = 0.0001, one-way ANOVA). Data are expressed as mean ± SEM. *P < 0.05; **P < 0.01; ***P < 0.001; n.s., not significant.

Results were compared to those obtained in age-matched WT animals. We found that, at 3 months of age, 3×Tg-AD mice did not exhibit any impairment in recognition and spatial memory, as assessed by NOR and MWM tests, respectively (Figures 1A–C). In particular, in the NOR test the preference index was comparable in 3×Tg-AD and age-matched WT mice (63.8 ± 1.7% and 65.7 ± 1.7%, respectively, *n* = 9 for each group; *P* = 0.40, one-way ANOVA; Figure 1A; exploration time: WT-3M mice, novel object (NO) 11.3 ± 1 s, familiar object (FO) 5.9 ± 0.5 s; 3×Tg-AD-3M mice, NO 11.5 ± 2.6 s, FO 6.4 ± 1.3 s). Similarly, in the acquisition session of the MWM, all mice successfully acquired the task with latency to reach the platform decreasing progressively across training days [main effect of days: *F*_(3,48)_ = 34.13, *P* < 0.001, two-way RM ANOVA] and no significant differences between WT-3M and 3×Tg-AD-3M mice in all trials (*n* = 9 for each group; *P* = 0.73, two-way RM ANOVA; Figure 1B) were noted. In the probe test, the time spent in the target quadrant was similar in 3×Tg-AD-3M and WT-3M mice (28.6 ± 2.8 s vs. 27.0 ± 2.5 s, respectively, *P* = 0.66, one-way ANOVA; Figure 1C). Both groups spent significantly more time in the target quadrant compared to random quadrant occupancy [i.e., 15 s; WT-3M mice, *F*_(1,19)_ = 16.38, *P* = 0.0006; 3×Tg-AD-3M mice, *F*_(1,19)_ = 18.50, *P* = 0.0003, one-way ANOVA]. Memory deficits were, instead, manifested in 7-month-old 3×Tg-AD mice (3×Tg-AD-7M). In the NOR test, they showed a lower preference index than age-matched WT mice (53.2 ± 1.5% vs. 65.6 ± 1.4% in WT-7M mice; *n* = 8 for each group; *P* < 0.001, one-way ANOVA; Figure 1E; exploration time: WT-7M mice, NO 9.2 ± 1.2 s, FO 4.9 ± 0.7 s; 3×Tg-AD-7M, NO 6.2 ± 1.5 s, FO 5.5 ± 1.3 s). In the acquisition session of the MWM, all mice displayed decreased latency to reach the hidden platform over training days [main effect of days: *F*_(3,42)_ = 14.72, *P* < 0.001, two-way RM ANOVA, but 3×Tg-AD-7M mice took longer time to find the platform than WT-7M mice (*n* = 8 for each group; *P* = 0.009, two-way RM ANOVA; Figure 1F). In the probe test, 3×Tg-AD-7M mice explored the target quadrant less than controls (17.4 ± 3.5 s vs. 27.0 ± 2.5 s in WT-7M mice; *P* = 0.032, one-way ANOVA; Figure 1G). Finally, WT-7M mice spent significantly more time in the target quadrant compared to random quadrant occupancy while 3×Tg-AD-7M mice failed to do so [WT-7M mice, *F*_(1,18)_ = 16.17, *P* = 0.0008; 3×Tg-AD-7M mice, *F*_(1,18)_ = 0.85, *P* = 0.36, one-way ANOVA].

As expected, behavioral data were paralleled by electrophysiological data showing a significant reduction of LTP at CA3–CA1 hippocampal synapses in brain slices from 3×Tg-AD-7M mice [34.37 ± 4.36% (*n* = 10 slices from 5 mice) vs. 78.85 ± 8.09% (*n* = 10 slices obtained from 5 WT-7M mice); *P* = 0.0001, one-way ANOVA; Figure 1H], whereas LTP was not significantly different in transgenic and WT mice at 3 months of age [65.11 ± 4.86% (*n* = 9 slices from 5 3×Tg-AD-3M mice) vs. 63.68 ± 10.74% (*n* = 9 slices from 6 WT-3M mice); *P* = 0.89, one-way ANOVA; Figure 1D]. Data reported above refer to analysis of fEPSP slope. A similar picture emerged when LTP was assessed by analyzing fEPSP amplitude (Supplementary Figures 1A,B). In agreement with our previous result (Leone et al., 2019). Western immunoblot experiments, performed with the 6E10 antibody recognizing human Aβ, revealed Aβ oligomers in hippocampal lysates of 3×Tg-AD-7M mice (Supplementary Figure 1C). A faint band was observed at the same molecular weight in tissues from 3×Tg-AD-3M.

Altogether these data indicate that, at 3 months of age, 3×Tg-AD mice do not show synaptic plasticity and memory deficits and, therefore, they are a suitable model of a pre-symptomatic AD stage to test our hypothesis.

### Anodal tDCS Fails to Enhance Recognition and Spatial Memory in 3×Tg-AD-3M Mice

**FIGURE 2:**
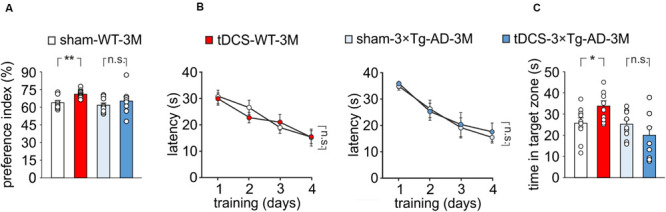
Effect of tDCS on memory in 3×Tg-AD-3M and WT-3M mice. **(A–C)** Memory was enhanced by tDCS in 3-month-old WT but not in 3×Tg-AD-3M mice, as shown by: **(A)** preference toward the novel object in NOR test (*n* = 9 sham-WT-3M mice vs. *n* = 10 tDCS-WT-3M mice, *P* = 0.001; *n* = 9 sham-3×Tg-AD-3M mice vs. *n* = 8 tDCS-3×Tg-AD-3M mice, *P* = 0.42, one-way ANOVA); **(B)** latency to reach the platform in the training phase of the MWM test (*n* = 10 sham-WT-3M mice and *n* = 9 tDCS-WT-3M mice, *P* < 0.001; *n* = 9 sham-3×Tg-AD-3M mice and *n* = 9 tDCS-3×Tg-AD-3M mice, *P* < 0.001, two-way RM ANOVA across training days) and **(C)** time spent in the target quadrant during probe test (sham-WT-3M mice vs. tDCS-WT-3M mice, *P* = 0.029; sham-3×Tg-AD-3M mice vs. tDCS-3×Tg-AD-3M mice; *P* = 0.24, one-way ANOVA). Data are expressed as mean ± SEM. *P < 0.05; **P < 0.01; n.s., not significant.

We then compared memory performances of 3×Tg-AD-3M and age-matched WT mice subjected to a protocol of triple tDCS or sham stimulation. Consistently with our previous findings (Podda et al., 2016), WT mice subjected to tDCS showed a greater preference toward the novel object than sham-stimulated mice [preference index: 70.7 ± 1.1% (*n* = 10) and 63.5 ± 1.8% (*n* = 9), respectively, *P* = 0.001, one-way ANOVA; Figure 2A]. As expected from data reported above, sham-3×Tg-AD-3M mice showed intact recognition memory [preference index: 61.0 ± 2.1% (*n* = 9), *P* = 0.36 vs. sham-WT-3M mice, one-way ANOVA; Figure 2A]. Of note, preference for the novel object was not increased by tDCS in 3×Tg-AD-3M mice [preference index: 64.6 ± 4.3% (*n* = 8), *P* = 0.42 vs. sham-3×Tg-AD-3M mice (*n* = 9) one-way ANOVA; Figure 2A]. Similar results were obtained with MWM, as shown in Figures 2B,C. In the acquisition session of the MWM, all mice successfully acquired the task with latency to reach the platform decreasing progressively across training days [WT-3M mice: main effect of days: *F*_(3,51)_ = 23.85, *P* < 0.001, two-way RM ANOVA; 3×Tg-AD-3M mice: main effect of days: *F*_(3,48)_ = 21.33, *P* < 0.001, two-way RM ANOVA; Figure 2B], with no significant differences between sham and tDCS in both groups (WT-3M mice: *P* = 0.81; 3×Tg-AD-3M: *P* = 0.71, two-way RM ANOVA). In the probe test, WT mice, but not 3×Tg-AD-3M mice, showed improvement following tDCS [tDCS-WT-3M, 33.5 ± 2.5 s (*n* = 9) vs. 25.5 ± 2.5 s (*n* = 10) sham-WT-3M; *P* = 0.029, one-way ANOVA; tDCS-3×Tg-AD-3M, 19.8 ± 3.9 s (*n* = 9) vs. 24.9 ± 2.2 s (*n* = 9) sham-3×Tg-AD-3M; *P* = 0.24, one-way ANOVA; Figure 2C).

### Anodal tDCS Fails to Enhance LTP in 3×Tg-AD-3M Mice

**FIGURE 3:**
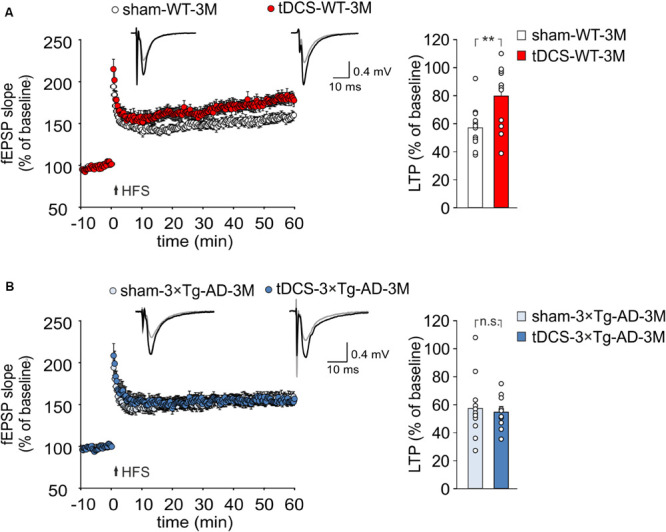
tDCS differentially impacts hippocampal LTP in 3×Tg-AD-3M and WT mice. **(A,B)** Time course of LTP at CA3-CA1 synapses induced by HFS delivered at time 0 (arrow). Results are expressed as percentages of baseline fEPSP slope (= 100%). Insets show representative fEPSPs at baseline (gray line) and during the last 5 min of LTP recording (black line). Bar graphs compare LTP observed during the last 5 min of recording. **(A)** Slices obtained from tDCS-WT-3M mice (*n* = 12 slices from 7 mice) showed enhanced LTP compared to sham-WT-3M mice (*n* = 12 slices from 9 mice, *P* = 0.007, one-way ANOVA). **(B)** tDCS failed to enhance LTP in 3×Tg-AD-3M mice (*n* = 10 slices from 5 tDCS mice; *n* = 12 slices from 5 sham mice, *P* = 0.71; one-way ANOVA). Data are expressed as mean ± SEM; **P < 0.01; n.s., not significant.

TDCS effects on memory have been reportedly associated to increased hippocampal LTP (Podda et al., 2016; Yu et al., 2019). We therefore asked whether the behavioral unresponsiveness to tDCS of 3×Tg-AD-3M mice was associated to the lack of tDCS effects on synaptic plasticity. FEPSP slope was measured in the CA1 area after standard HFS of Schaffer collaterals and LTP was studied in slices from WT and 3×Tg-AD-3M mice subjected to tDCS or sham stimulation. Sixty min after HFS, slices from tDCS-WT mice showed significantly greater LTP than slices from sham-WT mice [79.65 ± 6.58% (*n* = 12 slices from 7 tDCS mice) vs. 57.0 ± 4.4% (*n* = 12 slices from 9 sham mice); *P* = 0.007, one-way ANOVA; Figure 3A and Supplementary Figure 2A]. Conversely, LTP was not increased by tDCS in 3×Tg-AD-3M mice [54.71 ± 3.89% (*n* = 10 slices from 5 tDCS mice) vs. 57.49 ± 6.23% (*n* = 12 slices from 5 sham mice); *P* = 0.71, one-way ANOVA; Figure 3B and Supplementary Figure 2B], demonstrating that in these mice the cellular correlate of memory is also resistant to the boosting action of tDCS.

### Molecular Determinants of Plasticity Are Resistant to tDCS Boosting Effects in 3×Tg-AD-3M Mice

The above reported results demonstrate that, before the AD-like phenotype is manifested, 3×Tg-AD mice – despite normal memory and hippocampal LTP – exhibit decreased responsiveness to the boosting action of tDCS. The reduced response to tDCS might result from initial dysfunction of the molecular pathways underlying plasticity that are challenged by tDCS.

To test this hypothesis, we performed molecular analyses on hippocampi and blood samples from WT and 3×Tg-AD-3M mice subjected to tDCS or sham stimulation. Our analyses were focused on known upstream mechanisms of tDCS action, such as Ca^2+^-dependent phosphorylation of CREB at Ser133 and of CaMKII at Thr286, and a pivotal downstream effector, i.e., the neurotrophin BDNF (Podda et al., 2016; Kim et al., 2017; Paciello et al., 2018; Stafford et al., 2018; Barbati et al., 2019).

**FIGURE 4:**
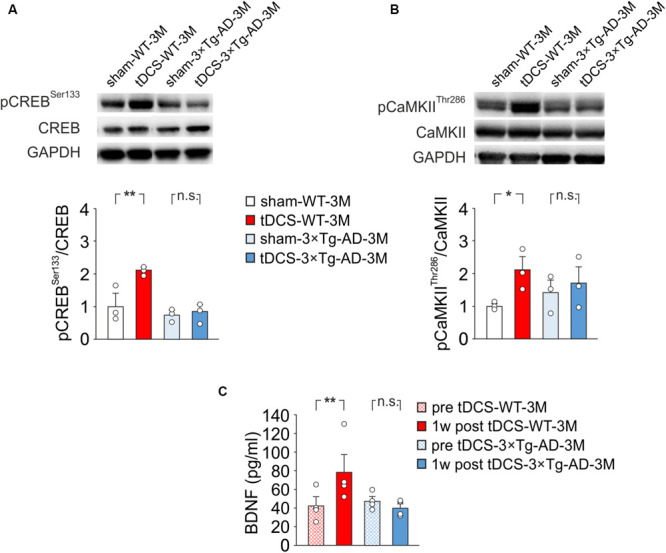
Molecular changes in 3×Tg-AD-3M and WT-3M mice following tDCS. Representative immunoblots revealed increased pCREB^*Ser133*^ **(A)** and pCaMKII^*Thr286*^ **(B)** following tDCS in WT-3M mice but not in 3×Tg-AD-3M mice. Bar graphs in the lower panel show results of densitometric analyses on all samples (*n* = 3 mice for each group; pCREB^*Ser133*^, *P* = 0.003 tDCS-WT-3M vs. sham-WT-3M; *P* = 0.77 tDCS-3×Tg-AD-3M vs. sham-3×Tg-AD-3M; pCaMKII^*Thr286*^, *P* = 0.045 tDCS-WT-3M vs. sham-WT-3M; *P* = 0.58 tDCS-3×Tg-AD-3M vs. sham-3×Tg-AD-3M two-way ANOVA, Bonferroni *post hoc*) normalized to both the corresponding total protein levels and GAPDH. **(C)** Plasma BDNF levels were measured before (pre) and 1 week after (1 w post) tDCS. BDNF was increased by tDCS in WT mice (*P* = 0.031, one-way RM ANOVA) but not in 3×Tg-AD-3M mice (*P* = 0.12, Friedman RM ANOVA on Ranks) (*n* = 4 mice for each group). Data are expressed as mean ± SEM. *P < 0.05; **P < 0.01; n.s., not significant.

Our previous observations indicated that tDCS induced CREB activation in the hippocampus 2 h after stimulation (Podda et al., 2016). Accordingly, immunoblot analyses revealed that, 2 h after the end of the last tDCS session, hippocampi of WT mice (*n* = 3) showed increased levels of pCREB^*Ser133*^ [+110% vs. sham-WT-3M mice (*n* = 3), *P* = 0.003; two-way ANOVA, Bonferroni *post hoc*; Figure 4A] and pCaMKII^*Thr286*^ (+109% vs. sham-WT-3M mice, *P* = 0.045 two-way ANOVA, Bonferroni *post hoc*; Figure 4B]. Intriguingly, these post-translational modifications were not observed in 3×Tg-AD-3M mice following tDCS (pCREB^*Ser133*^: +11% vs. sham-3×Tg-AD-3M mice; *P* = 0.77; pCaMKII^*Thr286*^: +19% vs. sham-3×Tg-AD-3M mice; *P* = 0.58; two-way ANOVA, Bonferroni *post hoc*; *n* = 3 mice each group; Figures 4A,B).

We previously reported that enhanced pCREB^*Ser133*^ following tDCS increases BNDF expression in the hippocampus by epigenetic regulation of *Bdnf* promoter I (Podda et al., 2016), and similar results were observed in auditory and motor cortices exposed to tDCS (Paciello et al., 2018; Barbati et al., 2019). We, therefore, hypothesized that tDCS could differentially impact BNDF expression in WT-3M and 3×Tg-AD-3M mice. Given that changes of brain BDNF expression are reflected in blood (Laske et al., 2006; Brunoni et al., 2015), we asked whether assessment of changes in plasma BDNF following tDCS could be a reliable biomarker of altered brain plasticity in AD. Blood samples used for BDNF testing were collected from each studied mice before starting the tDCS and 1 week after the completion of the tDCS protocol. This time point was chosen based on the results of a meta-analysis showing that increased plasma BDNF levels are more frequently observed some days after different protocols of non-invasive brain stimulation (NIBS) than soon after (Brunoni et al., 2015), and our previous studies demonstrated enhanced BDNF expression in the hippocampus 1 week after tDCS (Podda et al., 2016).

Remarkably, we found that plasma BNDF levels were significantly increased after tDCS in WT-3M (78.5 ± 20.2 vs. 42.3 ± 9.9 pg/ml pre-stimulation, *n* = 4 mice; *P* = 0.031, one-way RM ANOVA) but not in 3×Tg-AD-3M mice (40.1 ± 4.9 vs. 47.8 ± 5.0 pg/ml pre-stimulation, *n* = 4 mice; *P* = 0.12, Friedman RM ANOVA on Ranks; Figure 4C).

Our findings indicate that in 3×Tg-AD-3M mice molecular determinants of plasticity such as CREB, CaMKII and BDNF are resistant to the boosting effects of tDCS. More importantly, the early impairment of molecular machinery underlying synaptic plasticity and memory in 3×Tg-AD-3M mice can be detected by BDNF blood testing following tDCS.

## Discussion

AD is the most common form of dementia in elderly, characterized by a severe and progressive cognitive decline. So far, no effective treatments have been identified, but accumulating evidence suggests that therapeutics might work best if started at an early disease stage. The preclinical and prodromal phases of AD are considered promising time-windows for disease-modifying interventions (Galluzzi et al., 2016; Joe and Ringman, 2019). Therefore, early diagnosis is critical to successfully implement effective treatments.

The diagnosis of preclinical and prodromal AD is presently performed using cerebrospinal fluid analysis, neuroimaging investigations and neuropsychological testing (Lashley et al., 2018). Recently, graph theory analysis of brain connectivity from EEG signals combined with apolipoprotein E genotyping has been proposed to distinguish prodromal to AD from non-prodromal mild cognitive impairment (MCI) subjects (Vecchio et al., 2018). While these diagnostic approaches are valid and reliable, they cannot be employed for a wide ranging screening of persons at risk of AD, because they are invasive, expensive and require equipment and expertise usually only available in specialized hospitals.

Looking for an easy, non-invasive, low-cost and affordable method to screen populations at risk of AD, we investigated brain plasticity responses to tDCS in an AD mouse model before phenotype manifestation. This approach unveiled early electrophysiological and molecular dysfunction leading to the unresponsiveness of 3×Tg-AD-3M mice to tDCS boosting effects on memory, LTP and molecular determinants of synaptic plasticity.

Our data suggest that the assessment of plasticity-related molecular biomarkers before and after tDCS could represent a novel approach to predict AD onset and progression. Of particular relevance for a translational point of view, are the differential effects of tDCS on plasma BDNF levels.

In this study 3-month-old 3×Tg-AD mice were used as a model of preclinical AD. These mice showed normal memory, as their performance in the NOR and MWM tests was similar to that of age-matched WT mice. At 3 months of age LTP values were also comparable in WT and transgenic mice. Impaired memory and LTP were, instead, observed in AD mice at 7 months of age. Although a certain degree of 3×Tg-AD mouse model heterogeneity has been reported regarding the onset and progression of cognitive deficits, the timeline of the AD phenotype, in our experimental conditions, is in agreement with literature (Chakroborty et al., 2019; Joseph et al., 2019).

The NIBS techniques have recently gained considerable attention as promising approaches to slow the progression of AD (Rajji, 2019a). Despite encouraging data, conflicting results have been reported so far, likely due to different study designs, patient selection criteria, populations, or sample sizes, therefore, the efficacy of NIBS in AD is still uncertain (Rajji, 2019b). As far as animal models are concerned, tDCS failed to rescue learning and memory deficits in 3×Tg-AD mice when the phenotype is manifested (i.e., >6 months of age) (Gondard et al., 2019).

We propose to use tDCS in AD differently, namely, as a tool to probe and challenge plasticity pathways in the pre-symptomatic phase of the disease in order to unveil their earliest alterations.

Indeed, several studies, including our own, indicated that molecular determinants of plasticity and, particularly, the neurotrophin BDNF, are engaged and boosted by anodal tDCS, leading to enhanced plasticity and memory (Rohan et al., 2015; Podda et al., 2016; Kim et al., 2017; Cocco et al., 2018; Paciello et al., 2018; Stafford et al., 2018; Barbati et al., 2019; Kronberg et al., 2020).

Consistently, we found that 3-month-old WT mice, subjected to a daily session of anodal tDCS for three consecutive days, showed enhanced hippocampus-dependent recognition and spatial memory as assessed by NOR and MWM tests as well as enhanced LTP – the cellular underpinning of memory (Bliss and Collingridge, 1993). Interestingly enough, none of these effects was seen in 3×Tg-AD-3M mice.

We, therefore reasoned that the lack of tDCS effects on LTP and memory in 3×Tg-AD-3M mice might be due to the unsuccessful recruitment of plasticity-related pathways. We previously identified the signaling cascade engaged by tDCS in the hippocampus, including increased CREB phosphorylation at Ser133 that triggers epigenetic modifications relying on CREB binding to the *Bdnf* promoter I and recruitment of the histone acetyltranferase CREB-binding protein leading to enhanced acetylation at lysine 9 on *Bdnf* promoter I and increased BDNF expression. Blockade of H3 acetylation as well as of BDNF-specific TrkB receptors hindered tDCS effects on LTP and memory. Collectively, data summarized above suggested a causal link among the tDCS-induced increases in: (i) CREB phosphorylation; (ii) BDNF expression; (iii) synaptic plasticity; and (iv) memory (Podda et al., 2016). It has also been hypothesized that molecular events underlying tDCS effects are initiated by increased Ca^2+^ signaling via NMDAR and voltage-gated calcium channel activation (Pelletier and Cicchetti, 2014; Rohan et al., 2015). Indeed, Ca^2+^-dependent intracellular responses observed following tDCS include increased phosphorylation of CREB and CaMKII along with nitric oxide synthase activation (Kim et al., 2017; Cocco et al., 2018; Barbati et al., 2019). In keeping with these data, our Western immunoblot analyses showed enhanced pCREB^*Ser133*^ and pCaMKII^*Thr286*^ in tDCS-WT-3M mice. Of relevance, the lack of tDCS effects on LTP and memory in 3×Tg-AD-3M mice was paralleled by its inability to enhance pCREB^*Ser133*^ and pCaMKII^*Thr286*^, indicating that these differential response could serve as novel AD biomarker. Investigating the role of Ca^2+^ signal dysregulation in the tDCS ineffectiveness on LTP and memory in 3×Tg-AD-3M mice was beyond the scope of this research. However, it is worth mentioning that enhanced Ca^2+^ signaling has been reported in the earliest stages of the disease in mouse AD models (Del Prete et al., 2014; Chakroborty et al., 2019) and it has also been observed in cells from familial AD patients (Nelson et al., 2010). Furthermore, convergent evidence indicates Ca^2+^ dyshomeostasis within synaptic compartments as an early and critical factor in driving synaptic pathophysiology, leading to cognitive impairment in AD (Whitcomb et al., 2015).

The main purpose of our study was to identify an early and easy-to-detect AD biomarker potentially translatable to clinical application. Of course, molecular changes only occurring in the brain would not meet these requirements; therefore, we looked for biomarkers available in the circulating blood. Changes in pCREB and pCaMKII levels in the brain might be paralleled by similar changes in neuron-derived exosomes isolated from circulating blood, which is a promising though still experimental approach (Shi et al., 2016; Badhwar and Haqqani, 2020) we are planning to implement in future studies. Instead, we focused on a much simpler and cheaper approach, based on plasma BDNF level assessment by ELISA (Naegelin et al., 2018), which could be employed in any laboratory performing blood sample testing and therefore, widely accessible to any population. As already mentioned, enhanced BDNF expression in hippocampal lysates was demonstrated in our previous study following tDCS. Although different organs may contribute to determine plasma BDNF levels, several evidences suggest that changes in blood BDNF levels may reflect changes occurring in the brain. Indeed, changes in blood BDNF levels have been associated with a number of neurological diseases including AD (Laske et al., 2006), and they have also been more frequently reported days or weeks after stimulation following tDCS in different clinical conditions or experimental models (Brunoni et al., 2015). We, therefore, compared plasma BDNF levels before and 1 week after tDCS and found that they were significantly increased in WT but not in 3×Tg-AD-3M mice. Investigating the specific contribution of hippocampus vs. other cortical and subcortical areas underneath the stimulating electrode to plasma BDNF levels as well as its different forms (i.e., mature vs. pro-BDNF) was beyond the scope of this paper. Similarly, our study did not address the role of BDNF in AD pathophysiology.

Instead, our novel finding provides a peripheral biomarker of covert neuroplasticity impairment that could be detected in blood samples and easily translated to clinical use. The non-invasiveness and lack of adverse effects of tDCS (Antal et al., 2017) support future longitudinal studies in patient cohorts at risk of AD including elderly people diagnosed for amnestic MCI or those with genetic risk factors. In summary, our study unravels the unresponsiveness of neuroplasticity mechanisms in the hippocampus to boosting stimuli in a pre-AD stage. The combined use of a non-invasive method such as tDCS and plasma BDNF level assessment before and after treatment appears a novel promising approach to detect synaptic dysfunction far earlier than the appearance of any clinical signs. Although our findings still need to be validated in humans, they indicate a very promising perspective for large population analyses of subjects at risk to develop AD, with far reaching implications for both a personalized approach to AD patients and public health.

## Data Availability Statement

The raw data supporting the conclusions of this article will be made available by the authors, without undue reservation, to any qualified researcher.

## Ethics Statement

The animal study was reviewed and approved by the Ethics Committee of the Catholic University and Italian Ministry of Health.

## Author Contributions

CG and MP conceived the study and supervised the work. SC, VL, PR, and GA performed the electrophysiological experiments. MR and AM performed the behavioral experiments. SF performed the ELISA experiments. KG and SF performed the WB experiments. DL performed the analysis of Aβ oligomers. MP and CG wrote the manuscript. All authors contributed to the article and approved the submitted version.

## Conflict of Interest

The authors declare that the research was conducted in the absence of any commercial or financial relationships that could be construed as a potential conflict of interest.
