# Supplementary material for: Plasma BDNF Levels Following Transcranial Direct Current Stimulation Allow Prediction of Synaptic Plasticity and Memory Deficits in 3×Tg-AD Mice
Source: Front Cell Dev Biol. 2020 Jul 3;8:541. doi: 10.3389/fcell.2020.00541 (PMC7349675; doi:10.3389/fcell.2020.00541)
Supplement: Supplementary file 1 [file Data_Sheet_1.docx]

Supplementary Material

**Supplementary Materials and Methods**

**Western immunoblot analysis of Aβ oligomers**

Western immunoblot analysis was performed as previously with minor modifications (Li Puma et al., 2019). Hippocampal tissues from WT and 3×Tg-AD mice (n=3 for each group) were homogenized in ice-cold RIPA buffer (Pierce) in the presence of phosphatase and protease inhibitors (Sigma) and sonicated for 10 minutes. Protein concentrations were determined by Bradford protein assay (Biorad) and equal amounts of proteins (30 μg) were diluted NuPAGE LDS sample buffer 4× and separated on 4%-20% Tris-glycine polyacrylamide gels (Biorad) in non-denaturing and non-reducing conditions. Proteins were then transferred onto 0.2 µm nitrocellulose membranes (Amersham Biosciences) that were incubated overnight at 4 °C with the mouse monoclonal antibodies 6E10 (1:1000; Biolegend) and tubulin (1:5000; Abcam). The next day membranes were revealed with HRP-conjugated secondary antibodies (Cell Signaling Technology) using the westar eta c ultra 2.0 (Cyanagen) and documented by using UVItec Cambridge Alliance. Molecular weights for immunoblot analysis were determined using Low Range Rainbow Molecular Weight Markers (GE Healthcare Life Sciences).

**Supplementary figures**


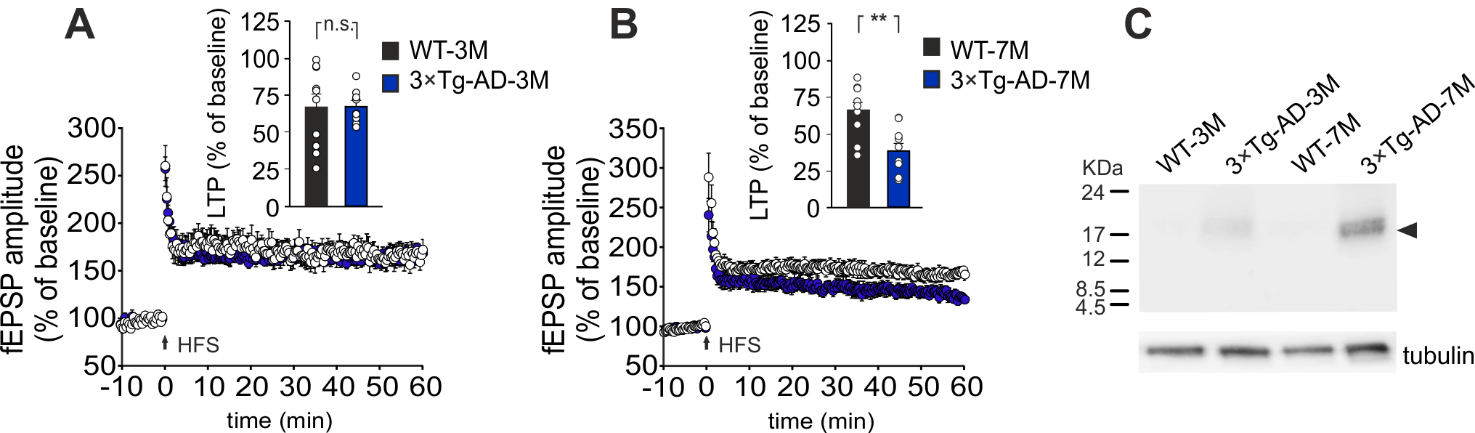


**Supplementary Figure 1. LTP and Aβ accumulation in hippocampi of 3-month- and 7-month-old 3×Tg-AD and age-matched WT mice. (A,B)** Time course of LTP at CA3-CA1 synapses induced by HFS (4 trains of 50 stimuli at 100 Hz for 500 ms repeated every 20 seconds) delivered at time 0 (arrow). Results are expressed as percentages of baseline fEPSP amplitude (=100%). Insets show bar graphs comparing LTP observed during the last 5 min of recording. Compared to aged-matched WT mice 3×Tg-AD-3M mice showed similar LTP (n=9 slices from 5 3×Tg-AD-3M mice; n=10 slices from 6 WT-3M mice; P=0.97, one-way ANOVA), whereas 3×Tg-AD-7M mice showed significant decreases in LTP (n=10 slices from 5 3×Tg-AD-7M mice; n=10 slices from 5 WT-7M mice, P=0.001, one-way ANOVA). **(C)** Representative Western immunoblot showing the presence of Aβ oligomers (indicated by the arrowhead) clearly detectable only in hippocampal tissue isolated from 3×Tg-AD-7M mice. Age-matched WT mice were used as negative controls. Data are expressed as mean ± s.e.m.; **P<0.01; n.s., not significant.

**
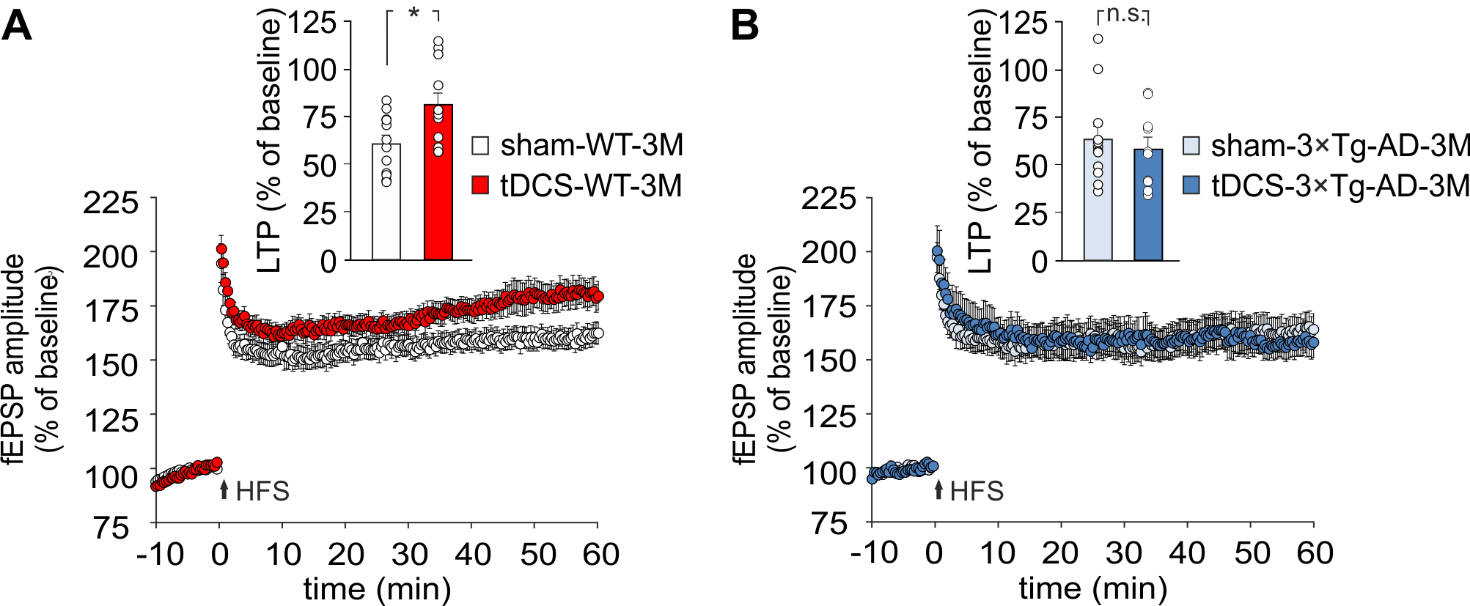
**

**Supplementary Figure 2. tDCS differentially impacts hippocampal LTP in 3×Tg-AD-3M and WT-3M mice. (A, B**) Time course of LTP at CA3-CA1 synapses induced by HFS (4 trains of 50 stimuli at 100 Hz for 500 ms repeated every 20 seconds) delivered at time 0 (arrow). Results are expressed as percentages of baseline fEPSP amplitude (=100%). Insets show bar graphs comparing LTP observed during the last 5 min of recording. **(A)** Slices obtained from tDCS-WT-3M mice (n=12 slices from 7 mice) showed enhanced LTP compared to sham-WT-3M mice (n=12 slices from 9 mice, P=0.014, one-way ANOVA). **(B)** TDCS failed to enhance LTP in 3×Tg-AD-3M mice (n=10 slices from 5 tDCS-3×Tg-AD-3M mice; n=12 slices from 5 sham-3×Tg-AD-3M mice, P=0.59; one-way ANOVA). Data are expressed as mean ± s.e.m; *P<0.05; n.s., not significant.

**Supplementary Table 1. Antibodies used in this study.**

| Antibody | #Catalog | Company | Experiment | Dilution | Host |
| --- | --- | --- | --- | --- | --- |
| CamKIIα | Sc-32288 | Santa Cruz | WB | 1:1000 | Mouse |
| phospho CamKII Thr286 | 12716 | Cell Signaling | WB | 1:1000 | Rabbit |
| CREB | MA1-083 | Thermo Fisher | WB | 1:500 | Mouse |
| phospho CREB Ser133 | 9198 | Cell signaling | WB | 1:1000 | Rabbit |
| GAPDH | Ab8245 | Abcam | WB | 1:5000 | Mouse |
| Anti-rabbit IgG HRP-linked | 7074 | Cell signaling | WB | 1:4000 | Goat |
| Anti-mouse IgG HRP-linked | 7076 | Cell signaling | WB | 1:4000 | Horse |
| Human Aß | 803001 | BioLegend | WB | 1:1000 | Mouse |
| Tubulin | Ab7291 | Abcam | WB | 1:5000 | Mouse |

Abbreviation: WB, Western immunoblot.
